# Editorial: Impacts of common geriatric syndromes and their interaction with chronic diseases on health

**DOI:** 10.3389/fmed.2022.1029246

**Published:** 2022-09-27

**Authors:** Jinhui Wu, Lin Kang, Ming Yang, Andrea P. Rossi

**Affiliations:** ^1^National Clinical Research Center for Geriatrics (WCH), West China Hospital, Sichuan University, Chengdu, China; ^2^Center of Gerontology and Geriatrics, West China Hospital, Sichuan University, Chengdu, China; ^3^Department of Geriatrics, Peking Union Medical College Hospital (CAMS), Beijing, China; ^4^Geriatrics Division, Department of Medicine, Healthy Aging Center Treviso, Treviso, Italy

**Keywords:** sarcopenia, frailty, functional disability, malnutrition (MeSH), geriatric syndromes, chronic diseases, innovative tools

One of the most critical tasks of geriatric care is maintaining and improving the function and health of the elderly (1). In addition to chronic diseases, geriatric syndromes (e.g., sarcopenia, frailty, functional disability, and malnutrition) substantially affect elderly health (2). Geriatric syndromes may interact with chronic diseases but their effects on elderly health are not fully understood (3).

This Research Topic includes not only original studies but also narrative reviews and systematic reviews, which provide updated knowledge on important geriatric syndromes and their interactions with chronic diseases on elderly health.

## Relevant new findings in this series

### Frailty

Setiati et al. conducted a multi-center cross-sectional study in Indonesia in 2020. Among the included 908 individuals, 167 (18.70%) had frailty. The authors reported that frailty was related to functional dependence, malnutrition risk, depression, a history of falls, a history of hospitalization, and polypharmacy. Functional dependence defined by the Barthel Index was related to an approximately seven-fold higher risk of frailty, while depression was correlated with an approximately three-fold higher risk of frailty. Malnutrition risk or malnutrition was related to an increasingly higher risk for frailty. A history of falls or hospitalization was associated with frailty. Polypharmacy was associated with a two-fold higher risk for frailty.

Shi et al. investigated the frailty status among 1,246 older adults and its association with mortality. The authors reported that women were frailer than men. In the whole study population, frailty was associated with an increased risk of death. The authors also found that frailty posed a more significant mortality risk than age, which emphasizes the importance of the management of frailty.

Liang et al. evaluated the relationship between frailty, hospitalization, and costs in Chinese older patients. Frailty was determined by an electronic frailty index (eFI) according to routine electronic health records. Among the 42,821 individuals with enough data, 33.8% had eFI-defined frailty. The eFI-defined frailty was independently associated with prolonged hospital stays and in-hospital death. Besides, the costs were positively associated with the level of frailty.

Liu et al. provided new insights into the trajectory and correlation between intrinsic capacity (IC) and frailty. This longitudinal observational study recruited 230 older adults and followed them for 2 years. The prevalence of frailty and IC impairment was 23.0 and 67.9% in the baseline and increased to 41.8% and 81.6% at the end of the follow-up, respectively. The authors concluded that loss of IC and frailty overlapped and co-existed in their study population.

Meng et al. explored the potential biomarkers and possible biological mechanisms related to frailty. They used 246 serum samples from Chinese men to detect metabolic profiles by targeted high-performance liquid chromatography-tandem mass spectrometry, including amino acids, carnitines, acylcarnitines, and lysophosphatidylcholines (LPCs). Older adults with frailty had lower tryptophan and higher glycine levels than those without frailty. Several characterized biomarkers, such as LPC16:0, LPC18:2, and tryptophan, were associated with frailty.

Similarly, Pan et al. used metabolomics to find frailty biomarkers and putative frailty pathogenic processes. Liquid chromatography-mass spectrometry-based metabolomics was applied to evaluate 349 metabolites in Chinese serum samples. The results showed that intermediates of carbohydrate metabolism, saturated fatty acids, unsaturated fatty acids, and certain essential amino acids might be the candidate biomarkers for the early screening and assessment of frailty. The authors suggested the potential mechanisms of frailty as well.

Additionally, Chang et al. evaluated the association between videofluoroscopic swallowing study (VFSS) findings and the incidence of pneumonia in patients with dysphagia due to frailty. They recruited 190 patients and followed them for 3 months after the VFSS. During the follow-up, 47 (24.7%) patients had pneumonia. Airway penetration and aspiration were associated with an increased risk of pneumonia.

She et al. reviewed the literature focused on frailty in older patients with the 2019 coronavirus disease (COVID-19), including potential pathogenesis, evaluation methods, and intervention measures. The authors suggested that older patients with COVID-19 should have an assessment of their frailty status as soon as feasible. They offered a scientific reference for clinicians to comprehensively manage frailty in older patients with COVID-19, from assessment to diagnosis and intervention.

Dai et al. performed a systematic review to assess the predictive value of frailty in lung cancer. Six studies with 2,359 patients were enrolled. The results showed that pre-frail or frail patients were more likely to experience therapeutic toxicity and all-cause death. The authors concluded that frailty was a significant predictor of all-cause death and treatment toxicity in patients with lung cancer.

In a study of Chinese older individuals in the community, Zhang, Ruan et al. evaluated the association between physical frailty and health-related quality of life (HRQoL). Based on 429 individuals, the results showed that physical frailty was an independent determinant of overall HRQoL. This study provides new evidence on the association between frailty and HRQoL and emphasizes the importance of timely management of frailty.

### Malnutrition

Using a multi-stage random sampling method, Liu et al. built a large cohort of 86,514 older individuals living in the community in Xinjiang, China. Low BMI was defined as BMI < 18.5 kg/m^2^ (for individuals aged < 70 years) or BMI < 20 kg/ m^2^ (for individuals aged ≥ 70 years). The prevalence of low BMI varied by geography and age. As latitude declined and age grew, the prevalence of low BMI steadily increased. This finding needs to be validated in other populations.

By using an open-access database from the National Health and Nutrition Examination Survey, Yen et al. found that patients with different comorbidities were affected differently by alcohol drinking.

Lastly, based on a sample of 669 elderly hospitalized adults, Chen et al. found that albumin was negatively associated with inflammatory indexes. They also reported that patients with poor nutritional status were more prone to having pneumonia in older adults.

### Sarcopenia

Jing et al. explored the relationship of activities of daily living (ADL) disability with trunk muscle mass and quality metrics determined from computed tomography (CT) in older inpatients. They found that CT-derived muscle quality indicators, instead of muscle mass index, were associated with ADL disability. This finding reinforces the importance of muscle quality for assessing muscle health in sarcopenia research.

Chung et al. performed a meta-analysis with six studies to explore the prevalence of sarcopenia and its relationship to diabetes in community-dwelling individuals. The prevalence of sarcopenia, as determined by the Asian Working Group for Sarcopenia criteria, was 15.9% in people with diabetes and 10.8% in those without. Compared to people without diabetes, diabetics showed a noticeably greater risk of sarcopenia.

Kwack et al. proposed a new method to evaluate muscle mass. In a small cross-sectional study conducted in hemodialysis patients, the author's calculated psoas muscle (PM) index and normal-density psoas muscle (NPM) index using the whole PM area (-29–100 HU, as usual) or the normal-density PM area (35–100 HU, the new method) divided by height squared. The results suggested that in hemodialysis patients, the NPM index can be a practical predictor of muscle strength and physical performance. Muscle mass and muscle quality are both taken into account in this new method, and it deserves further investigation.

Huang and Wang reviewed the current evidence of sex hormones for treating sarcopenia. They also discussed the possible advantages of combining sex hormone treatments with exercise and dietary supplements.

Gao et al. reanalyzed the data from the China Health and Retirement Longitudinal Study (CHARLS) to explore the association between sarcopenia and depressive symptoms in Chinese older individuals. They included 7,706 participants aged ≥ 60 years. In the baseline, both probable sarcopenia and sarcopenia were positively associated with depressive symptoms. During the 3.7-year follow-up, 956 (20.6%) participants experienced depressive symptoms. Participants with probable sarcopenia or sarcopenia were more prone to having newly developed depressive symptoms. These findings provided new evidence that sarcopenia should be evaluated when fighting against depressive symptoms.

Lee et al. investigated the association between acute vertebral osteoporotic compression fractures (VOCF) and body composition in 461 older patients. The risk of acute VOCF was the highest in patients with sarcopenic obesity. Sarcopenic obesity was strongly linked with acute VOCF rate, particularly in women, according to multivariate analysis.

### Functional disability

Zhang, Liu et al. developed a novel screening tool named function impairment screening tool (FIST) for assessing physical function. Based on a cohort of 489 Chinese older adults, they proved that the FIST tool had good test-retest reliability and validity. However, its diagnostic value for screening functional impairment needs to be further validated in different populations.

Sun et al. reanalyzed the data of 13,959 adults aged ≥ 45 years from the CHARLS study and concluded that short or long sleep duration was associated with vision impairment. This finding is interesting and merits further research.

Moreover, Peng et al. reanalyzed the differentially expressed genes from two microarray data sets to explore prognostic gene expression signatures for age-related hearing loss. They concluded that distinct levels of hearing loss in presbycusis were correlated with different autophagy indicators and immunological microenvironments.

Lastly, Prell et al. showed that dizziness syndromes were completely different between older adults and their young counterparts. Younger individuals reported more severe dizziness-related distress, whereas older individuals reported experiencing dizzy symptoms for longer than younger individuals. Older individuals experienced fewer depressive symptoms and less anxiety than younger individuals. Younger individuals were more likely to be hospitalized and had more doctor consultations than older individuals.

### Cardiovascular disease

Some studies addressed cardiovascular diseases in older adults. For example, Zhang, Xiang et al. reviewed the evidence regarding the contribution of nursing to the treatment of cardiovascular diseases and concluded that nursing significantly improved the outcomes of heart failure and myocardial ischemia in older patients. This finding emphasized the importance of nursing in older patients with heart disease, which deserves further research in the future.

Additionally, in a prospective cohort study with 271 patients with acute non-ST-segment elevation myocardial infarction, Zhang, Wang et al. reported that elevated plasma myeloperoxidase level was related to high inflammatory status and a coronary events risk score.

Zhang, Xian et al. tested the diagnostic value of AMAZFIT^®^, a wearable electrocardiogram-recording device, for detecting arrhythmia in 291 older adults. This new tool appeared to have a considerable sensitivity and specificity for detecting atrial fibrillation, premature arterial contractions, and premature ventricular contractions when using a standard 12-lead ECG device as the gold standard.

Lastly, Miao et al. performed a systematic review to evaluate the possible value of serum uric acid (SUA) for predicting outcomes in patients with chronic heart failure. A total of 18 publications were included. The results showed that serum SUA was positively associated with an increased risk of all-cause mortality, cardiovascular mortality, and the composite of death or cardiac events.

### Osteoarthritis

In a meta-analysis involving 24 randomized controlled trials (RCTs) with 1,275 participants, Wang et al. evaluated the benefits of proprioceptive training for improving symptoms, function, and proprioception in patients with knee osteoarthritis (KOA). Proprioceptive training could substantially improve pain, stiffness, and physical function, and enhance joint position sense (JPS), muscular strength, mobility, and knee range of motion when compared to no intervention. Additionally, proprioceptive training provided better results in terms of JPS and mobility when compared to the control groups.

Traditional Chinese Yijinjing Qigong Exercise (YJJQE) is a popular exercise in China. Zhang, Guo et al. designed an RCT to assess the effectiveness of YJJQE for treating KOA. They randomly assigned 50 KOA patients to the YJJQE group (n = 25) or the stretching training exercise (STE) group (n = 25). For a period of 12 weeks, each participant had two 40-min exercise sessions (YJJQE or STE) twice per week. They found no difference between groups with regards to the primary outcome, the Western Ontario and McMaster Universities Osteoarthritis Index Scale score. However, YJJQE seemed to be related to an improvement in psychological wellbeing.

### Miscellaneous

Huang, Jia et al. reviewed recent findings on *Helicobacter pylori* (H. pylori) diagnosis, management, and treatment in older adults, highlighting the differences between older patients and their younger counterparts. The use of routine therapy in older patients was constrained by health issues, such as comorbidities and polypharmacy. However, eradication treatment may still be advantageous for older patients. New methods, such as complementary and dual therapies, may also be used to treat elderly people with H. pylori infection.

In a systematic review with 29 RCTs with 1,994 participants, Zhang, Ou et al. summarized current evidence regarding transcutaneous electrical acupoint stimulation (TEAS) for preventing postoperative cognitive dysfunction. The results demonstrated that the perioperative use of TEAS reduced postoperative cognitive dysfunction rates and protected early postoperative cognition.

To describe the impact of new androgen receptor axis-target (ARAT) drugs on diarrhea and constipation, Xiong et al. performed a systematic review including 13 trials with 15,117 participants. They compared four drugs (abiraterone, enzalutamide, apalutamide, and darolutamide) with placebo. These novel ARAT drugs were related to 1.3 times increased risk of any-grade diarrhea. Patients with metastatic hormone-sensitive and castration-resistant prostate cancer were most likely to experience diarrhea when using abiraterone.

## Future research

This collection highlights new information and new trends regarding the epidemiology, dynamics, and management of common geriatric syndromes, such as frailty, sarcopenia, disability, and malnutrition, and their interaction with chronic diseases, with a particular focus on cardiovascular disease and osteoarthritis. Not surprisingly, most of the studies in this collection focused on frailty and sarcopenia, the two new “geriatric giants” and to a lesser extent, malnutrition (4). Unfortunately, other geriatric giants, such as cognitive impairment/dementia, delirium, and dysphagia, were less addressed in this collection and should be the topic for future special issues. Moreover, except for Huang, Wang et al. review of the literature, most of the included studies were observational and/or cross-sectional observations. In particular for sarcopenia and frailty, future research should concentrate on the treatment of those conditions with ad hoc clinical trials.

Here, we proposed some considerations for further research:

- Older adults with geriatric syndromes are more likely to suffer from chronic diseases (e.g., diabetes and cardiovascular disease) (5), while chronic diseases are associated with an increased number of geriatric syndromes (3). The cross-talk between different geriatric syndromes and chronic diseases and their synergistic effects on health require more future research.
- Although some authors argued that vascular diseases are key etiological of geriatric syndromes (6), the potential mechanism of geriatric syndromes remains unclarified. Further disentangling of the mechanisms of different organ aging may help us understand geriatric syndromes.
- More well-designed clinical trials are required to focus on the optimal interventions for preventing and treating geriatric syndromes. The interventions may include pharmacotherapy, lifestyle management, and rehabilitation/prerehabilitation. Some interventions (e.g., resistance training and vitamin D) that benefit both chronic diseases (e.g., osteoporosis) and geriatric syndromes (e.g., sarcopenia and frailty) may have priority. The study populations may focus on people who are prone to geriatric syndromes, such as the oldest old, institutional residents, and older patients in different clinical settings. The outcomes should include patient-reported outcomes and HRQoL.

## Author contributions

All authors listed have made a substantial, direct, and intellectual contribution to the work and approved it for publication.

## Funding

JW is funded by China National Key R&D Program (Nos. 2018YFC2002100 and 2018YFC2002103). LK is funded by National High-Level Hospital Clinical Research Funding (UH_PROMO_202201, APL221003101004008).

## Conflict of interest

The authors declare that the research was conducted in the absence of any commercial or financial relationships that could be construed as a potential conflict of interest.

## Publisher's note

All claims expressed in this article are solely those of the authors and do not necessarily represent those of their affiliated organizations, or those of the publisher, the editors and the reviewers. Any product that may be evaluated in this article, or claim that may be made by its manufacturer, is not guaranteed or endorsed by the publisher.
